# Effect of Adapted Ergometer Setup and Rowing Speed on Lower Extremity Loading in People with and Without Spinal Cord Injury

**DOI:** 10.3390/bioengineering12010075

**Published:** 2025-01-15

**Authors:** Ying Fang, Karen L. Troy

**Affiliations:** 1 Department of Physical Therapy, Rosalind Franklin University of Medicine and Science, North Chicago, IL 60064, USA; 2 Department of Biomedical Engineering, Worcester Polytechnic Institute, Worcester, MA 01609, USA; ktroy@wpi.edu

**Keywords:** FES rowing, spinal cord injury, knee loading, knee biomechanics

## Abstract

Background: Functional electrical stimulation-assisted rowing (FES rowing) is a rehabilitation exercise used to prevent disuse osteoporosis, which is common in people with spinal cord injury (SCI). However, its effect on bone loss prevention varied in SCI patients, potentially due to inconsistent loading. This study investigates the effect of ergometer setup and rowing speed on lower extremity loading during rowing. Methods: Twenty able-bodied participants and one participant with SCI rowed on an adapted ergometer with different speeds and setups. We calculated foot reaction force and knee moment for all participants, and tibiofemoral force for the rower with SCI. Results: Able-bodied rowers generated 0.22–0.45 body weight (BW) foot reaction forces, and a higher force was associated with a fast speed, forward seat position, and large knee range of motion (RoM). The rower with SCI had the greatest foot reaction force (0.39 BW) when rowing with a small knee RoM at a rear seat position, and the highest tibiofemoral force (2.23 BW) with a large knee RoM or at a rear seat position. Conclusions: Ergometer setup and speed both affect lower limb loading and should be further studied in more rowers with SCI. This can inform rehabilitation protocols to standardize ergometer configuration to improve bone health.

## 1. Introduction

People with spinal cord injury (SCI) experience severe bone loss in their lower limbs, leading to a 40% long-term fracture risk [1]. Physical activities reload the lower extremity and could potentially improve bone health in individuals with SCI [2,3,4]. A dose–response relationship has been observed with greater loading to the musculoskeletal system, leading to less bone loss for activities such as functional electrical stimulation-assisted (FES) cycling and electrical stimulation-induced standing [5,6,7].

FES rowing is one exercise intervention that is growing in popularity in SCI rehabilitation [8]. Previous studies reported inconsistent bone responses after training with FES rowing along with a wide range of foot reaction forces generated by rowers with SCI (0.15 to 0.46 body weight (BW)) [9,10,11,12,13,14,15]. These variable results, combined with observations that bone adapts to its mechanical loading environment, suggest that foot loading influences subsequent changes in lower extremity bone in FES rowing. Furthermore, it suggests that people who benefit from the exercise may row differently from those who do not. However, little is known about the potential factors that may influence force generation in FES rowing.

**Figure 1:**
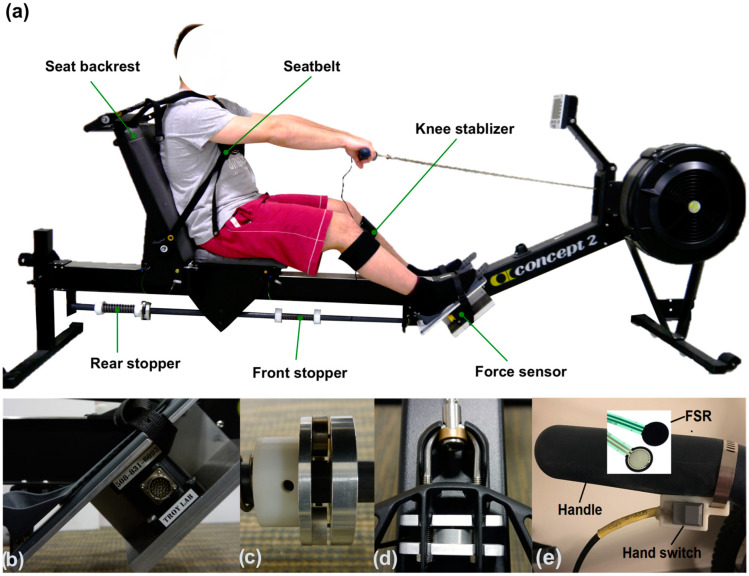
(**a**) The adapted rowing ergometer designed for functional electrical stimulation-assisted rowing, (**b**) a 6-axis force sensor under the right foot stretcher, (**c**) two single-axis load cells on the rear stopper, (**d**) one single-axis load cell on the handle, and (**e**) the hand switch of the functional electrical stimulator with an instrumented force-sensitive resistor.

In able-bodied rowing, both rowing speed and rowing experience affect the magnitude of foot reaction force, and consequently, joint loading. Elite rowers generate significantly higher foot reaction force and knee moment than novice or non-rowers [16]. Rowing speed influences force production in novice rowers, but not experienced rowers [16,17]. The design and setup of the ergometer also affect force production [18]. For example, increasing the height of the foot stretcher significantly reduced foot force and ankle, knee, and hip moments among elite rowers [19]. There are three major changes to a normal ergometer to make it suitable for FES rowing: (1) two stoppers are added on the seat rail to limit the anterior–posterior movement of the seat, which prevents knee hyperextension; (2) a seat backrest is added, and the rower wears a seatbelt for stabilization, so there is no trunk flexion/extension; (3) a knee stabilizer is placed between the legs during rowing to prevent the knees from separating apart (Figure 1). These changes add additional constraints to the activity and may alter rowing biomechanics. They also allow for more adjustments on the ergometer, which could affect force production by users with SCI.

Foot reaction force and joint loading during FES rowing are influenced by the amount of muscle force. FES-induced muscle contraction is different from voluntary muscle contraction: FES uses higher-frequency electrical pulses (20–40 Hz) compared to voluntary contraction (6–8 Hz) so all motor units contract simultaneously, causing a muscle to fatigue sooner [20,21]. Additionally, changes in muscle properties in people with SCI due to disuse, such as a decreased proportion of slow-twitch fibers, can also limit a muscle’s ability to produce constant forces [22]. In FES rowing, quadriceps and hamstrings are stimulated to induce knee extension and flexion, respectively, to allow users to accomplish the basic rowing movement on an adapted rowing ergometer [23].

Considering the recent finding that foot loading was lower than desired in FES rowing, the purpose of the study was to investigate the effect of rowing speed, knee range of motion (RoM), and seat position on lower extremity loading in a group of able-bodied adults and a single individual with SCI. Specifically, we focused on foot reaction force and knee extension moment in all participants and evaluated the tibiofemoral force in the participant with SCI. We hypothesized that rowing speed, knee RoM, and seat position would all affect the foot reaction force and knee moment produced by able-bodied rowers and the rower with SCI, as well as affect the peak tibiofemoral force of the rower with SCI.

## 2. Materials and Methods

### 2.1. Subjects

Ten male and ten female able-bodied adults (age: 26.5 ± 3.8 years, mass: 70.0 ± 14.8 kg, height: 1.7 ± 0.1 m) with no rowing experience or lower extremity injuries within the past six months, and one male participant with SCI (age: 29.0 years, mass: 75.0 kg, height: 1.7 m, injury years: 6 years, injury level: T-4 complete, FES rowing experience: 6 years) participated in this study. Each participant read and signed an informed consent document approved by the institutional review board before testing. Power analysis indicated that a sample size of 14 was enough to detect a within-subject difference in peak foot reaction force of 30 ± 20 N, with 80% power for conditions with different setups (α = 0.05).

### 2.2. Instrumentation

A ten-camera motion capture system (100 Hz; Vicon Motion Analysis Inc., Oxfordshire, UK) was used to collect 3D kinematic data. Reflective markers were placed bilaterally on the acromion processes, anterior superior iliac spines, great trochanters, medial and lateral epicondyles of the knee, medial and lateral malleoli, and the middle toes. Two markers were adhered to the seatback to represent the posterior superior iliac spines, which were blocked by the seatback during rowing. The above markers were used to define the trunk, pelvis, thighs, lower legs, and feet of the subject.

An adapted rowing ergometer (Concept2, model D, Morrisville, VT, USA; Figure 1), commonly used in clinical settings for FES rowing, was instrumented and used for all tests. A 6 degrees-of-freedom force sensor (1000 Hz; MC3A-1000lb, AMTI, Watertown, MA, USA) was mounted under the right foot stretcher of the ergometer to record the foot reaction forces and moments. A dummy block with the same geometry and weight of the sensor was mounted under the left foot stretcher to ensure both sides were symmetrical. Multiple single-axis load cells (0–200 kg; 3137_0, Phidgets Inc., Calgary, AB, Canada) were instrumented to measure hand force and rear stopper force (Figure 1, Table S1). During FES rowing, the SCI participant brought his own electrical stimulator (Odstock, Salisbury, UK) along with a hand switch to control the stimulation. The 4-channel stimulator generated electrical signals (no ramp, pulse width: 450 μs, frequency: 40 Hz) to contract the knee extensors and flexors: pressing the switch activates the quadriceps and releasing the switch activates the hamstrings [24]. The hand switch was instrumented with a force-sensitive resistor to measure when the switch was pressed (Figure 1). Signals from all sensors were amplified and filtered using a signal conditioner (GEN 5, AMTI, Watertown, MA, USA) and recorded within the VICON system and its Nexus software (version 2.5.0, Vicon Motion Analysis Inc., Oxfordshire, UK) simultaneously with the kinematic data.

### 2.3. Experimental Protocol

Upon arrival to the lab, able-bodied participants were instructed to row on the adapted ergometer and were given sufficient time to practice until a smooth movement was achieved. Then, individuals sat on the seat with both feet resting on the foot stretchers, and a researcher determined the location of the front and rear stoppers based on the participant’s knee angle. Specifically, the front stopper was adjusted such that the minimum knee angle was 45°, 70°, or 95°; the rear stopper was adjusted such that the maximum knee angle was 115°, 135°, 140°, or 165°. In this way, the seat position and knee RoM could be independently adjusted (Table S1). Participants rowed for 90 s at each of the 12 conditions that include three speeds (25, 35, and 40 SPM with a forward seat position and knee RoM of 70° and 90°), three knee RoM (70°, 90°, and 120° with a forward seat position at speed of 25 SPM and 35 SPM), and three seat positions (forward, middle, and rear, with knee RoM of 70° at speed of 25 SPM and 35 SPM).

The FES rowing test followed similar experimental procedures. The individual with SCI applied the electrodes and set up the stimulator himself, and the researcher determined the location of stoppers. To avoid fatigue, this participant tested 7 rowing conditions: a self-selected style and 6 out of the 12 conditions that were tested in able-bodied rowers. The 6 conditions were selected because they yielded the largest foot reaction forces in able-bodied participants. For both able-bodied and FES rowing, resistance on the ergometer was set at level 3, which is commonly used for FES rowing based on previous interviews with 4 rowers with SCI. Rowing speed was controlled by the real-time feedback on the monitor of the ergometer. Data were collected for 30 s starting from 30 s into the bout of rowing for each condition.

### 2.4. Data Analyses

The raw kinematics and force data were filtered using a low-pass fourth-order Butterworth filter at a cutoff frequency of 1 Hz. Joint angles were calculated based on joint centers and coordinate systems that were defined using ISB recommendations [25]. Specifically, the knee angle was defined as 180° when the knee was fully extended. Joint moments and forces were calculated with inverse dynamics using a recursive Newton–Euler approach and averaged for at least five rowing cycles under each condition [26]. The beginning of each cycle was defined when the seat was at the front-most position. We used customized code to perform all data analyses in Matlab (MathWorks, Natick, MA, USA). Peak foot reaction forces and knee extension moments were the primary outcomes of interest.

The tibiofemoral force of the rower with SCI was calculated using OpenSim 3.3 [27]. First, we developed a musculoskeletal model of the SCI participant by scaling a full-body generic model using experimental markers, and the shoulder, elbow, and wrist joints of the model were fixed [28]. The mass and inertial properties of each segment were initially scaled based on the participant’s mass [29], and then updated according to the anthropometric data specific to the SCI population [30]. Marker trajectories of each trial were applied to the scaled model to replicate experimentally measured kinematics. We assumed both legs generated symmetrical force and used the measured force under the right foot to estimate the force under the left foot. We also assumed all forces beyond the hip (e.g., hand force, seat force, rear stopper force) to be a resultant force applied at the pelvis (pelvis force), which was derived from dynamic analysis considering all measured external forces (Figure S1). We used static optimization to estimate muscle forces. Based on the electrode location, the quadriceps corresponded to the rectus femoris, vastus lateralis, and vastus medialis, and the hamstrings corresponded to the biceps femoris long head and semitendinosus in the model. The remaining muscles were enabled in the model with a fixed activation level of 0.01 to account for passive stiffness. Using the joint reaction analyses tool, we resolved tibiofemoral force considering muscle forces, external forces, and joint kinematics. Peak knee compressive tibiofemoral force was the primary outcome.

### 2.5. Statistical Analyses

For able-bodied rowing, repeated measures analyses of variance were used to examine the influence of rowing speed, knee RoM, and seat position on the peak foot reaction force and peak knee extension moment. If a main effect was significant, post hoc analysis was performed using pairwise *t*-tests with Bonferroni adjustments. All analyses were performed in SPSS (IBM SPSS Statistics 22, Chicago, IL, USA) with an alpha level of 0.05.

## 3. Results

All able-bodied participants successfully rowed at the required knee ranges of motion and speeds, and their peak foot reaction forces ranged from 0.22 to 0.45 BW across the 12 conditions with no significant difference between males and females.

The rower with SCI managed to row under four out of six conditions with acceptable variations from the target form and had difficulty completing a rowing cycle for the other two conditions (45–135°, 40 RPM and 45–165°, 25 RPM). His peak foot reaction force and peak compressive tibiofemoral force ranged from 0.26 to 0.39 BW and 1.21 to 2.23 BW, respectively, across all completed conditions. The rower with SCI’s self-selected form resulted in a 0.39 BW foot reaction force and a 1.83 BW peak compressive tibiofemoral force (Table 1).

### 3.1. Effect of Rowing Speed

**Figure 2:**
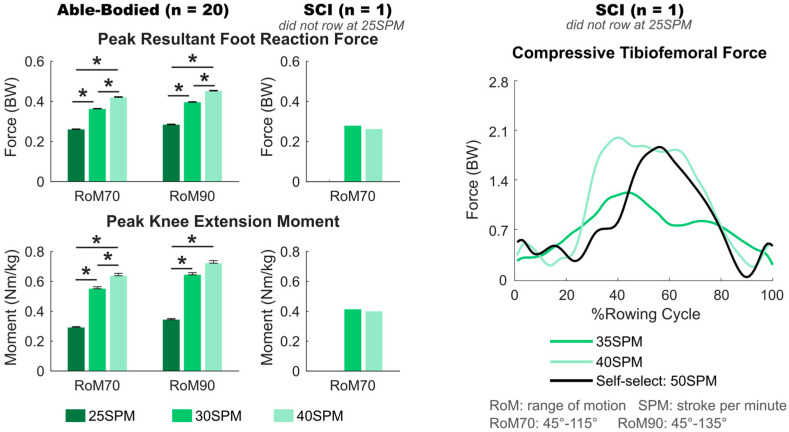
Peak resultant foot reaction force and peak knee extension moment of 20 able-bodied participants and the participant with spinal cord injury (SCI) rowing at different speeds with knee ranges of motion (RoM) of 70° and 90°, and compressive tibiofemoral force throughout the rowing cycle of the SCI participant. Error bars indicate standard errors, and * indicates significant differences with *p* < 0.05.

There was a significant speed effect on peak foot reaction force (*p* < 0.001) and peak knee extension moment (*p* < 0.001) in able-bodied rowers; faster speed resulted in a greater foot reaction force and knee moment at both 70° and 90° knee RoM. At 70° knee RoM, rowing at 40 SPM resulted in a significantly greater foot reaction force (*p* < 0.001) and knee moment (*p* ≤ 0.037), compared to rowing at 35 SPM or 25 SPM, and rowing at 35 SPM resulted in significantly greater foot reaction force (*p* < 0.001) and knee moment (*p* ≤ 0.037), compared to rowing at 25 SPM. At 90° knee RoM, the foot reaction force (*p* < 0.001) and knee moment (*p* < 0.001) were significantly different between 25 SPM and 35 SPM and between 25 SPM and 40 SPM conditions (Figure 2, Table 2).

For the rower with SCI, the peak foot reaction force decreased by 0.02 BW, peak knee moment decreased by 0.01 Nm/kg, and peak compressive tibiofemoral force increased by 0.75 BW when the speed increased from 35 SPM (actual speed: 31 SPM) to 40 SPM (actual speed: 42 SPM) with 70° knee RoM (Figure 2, Table 1).

### 3.2. Effect of Knee Range of Motion (RoM)

**Figure 3:**
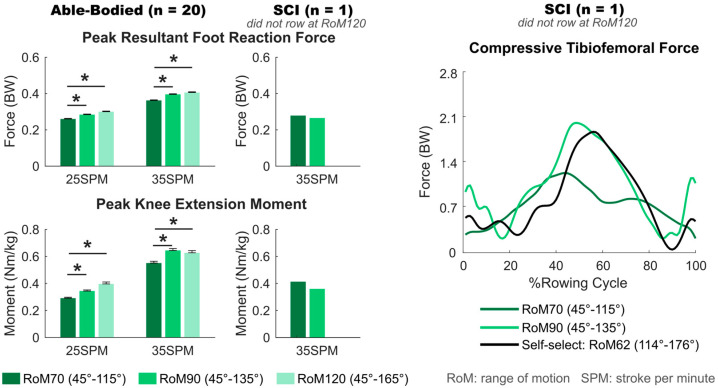
Peak resultant foot reaction force and peak knee extension moment of 20 able-bodied participants and the participant with spinal cord injury (SCI) rowing at different knee ranges of motion (RoM) at 25 and 35 strokes per minute (SPM), and compressive tibiofemoral force throughout the rowing cycle of the SCI participant. Error bars indicate standard errors, and * indicates significant differences with *p* < 0.05.

Knee RoM had a significant effect on the peak foot reaction force (*p* = 0.003) and peak knee extension moment (*p* = 0.012) in able-bodied participants. A larger knee RoM resulted in a greater foot reaction force and knee moment at both 25 SPM and 35 SPM. Regardless of rowing speed (25 SPM or 35 SPM), foot reaction force (*p* ≤ 0.009) and knee moment (*p* ≤ 0.037) were significantly lower during rowing with a 70° RoM compared to 90° or 120° RoM conditions (Figure 3, Table 3).

The rower with SCI had 0.01 BW smaller peak foot reaction force, 0.05 Nm/kg smaller peak knee moment, and 0.76 BW greater peak compressive tibiofemoral force when rowing with 90° (actual: 108°) compared to 70° (actual: 67°) knee RoM at 35 SPM (Figure 3, Table 1).

### 3.3. Effect of Seat Position

**Figure 4:**
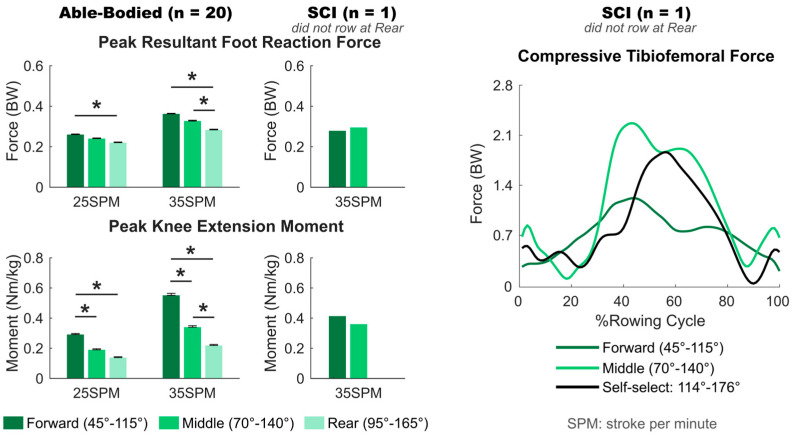
Peak resultant foot reaction force and peak knee extension moment of 20 able-bodied participants and the participant with spinal cord injury (SCI) rowing at different seat positions at 25 and 35 strokes per minute (SPM), and compressive tibiofemoral force throughout the rowing cycle of the SCI participant. Error bars indicate standard errors, and * indicates significant differences with *p* < 0.05.

In able-bodied rowers, seat position had a significant effect on the peak foot reaction force (*p* < 0.001) and peak knee extension moment (*p* < 0.001). A more rear seat position resulted in a smaller foot reaction force and knee moment at both 25 SPM and 35 SPM. Compared to a forward seat position, rowing with a rear seat position significantly decreased the peak foot reaction force (*p* ≤ 0.001) and peak knee moment (*p* ≤ 0.004) at both 25 SPM and 35 SPM rowing speeds. Rowing from a middle seat position resulted in a significantly decreased peak knee moment (*p* ≤ 0.007) and similar peak foot reaction force compared to a forward seat position at both 25 SPM and 35 SPM (Figure 4, Table 4).

The rower with SCI had a 0.01 BW greater peak foot reaction force, 0.05 Nm/kg smaller knee moment, and 1.02 BW greater peak compressive tibiofemoral force while rowing with a middle compared to a forward seat position at 35 SPM (Figure 4, Table 1).

## 4. Discussion

We investigated the effects of adapted rowing ergometer setup and speed on peak foot reaction force and knee extension moment in a group of able-bodied adults and a single participant with SCI. The hypotheses were supported that rowing speed, knee RoM, and seat position all affected lower extremity loading in able-bodied participants and the participant with SCI. In able-bodied individuals, having a forward starting position for the seat, combined with a fast speed and large knee RoM, was associated with the greatest foot reaction force and knee moment. However, this combination did not yield the greatest foot reaction force for the rower with SCI, who generated a greater foot reaction force by rowing with a smaller knee RoM and a more extended knee.

The able-bodied and SCI participants generated lower foot reaction forces than what has been previously reported. When novice able-bodied individuals rowed on a normal ergometer, the foot reaction force ranged from 0.53 to 0.98 BW [31,32]. Using the adapted ergometer in this study, all participants generated only half the foot reaction force: 0.22 to 0.45 BW for the able-bodied and 0.26 to 0.39 BW for the rower with SCI. This indicates that the adaptations made to the ergometer limited force production, and the maximum possible foot reaction force was less than 50% body weight for novice users. This amount of loading may not be sufficient to prevent bone loss, as was observed in some people with SCI following the FES rowing intervention [9,33]. With a normal ergometer, larger foot reaction forces can be achieved by increasing trunk extension at the end of the drive phase, ankle flexion at the beginning of the drive phase, and by having a larger hip RoM [17]. These strategies were limited when rowing on an adapted ergometer: the added seat back and seatbelt constrain both trunk and hip movement, and the front and rear stoppers limit ankle motion. Future research could focus on these design characteristics of the rowing ergometer and come up with solutions to allow trunk extension and flexion while also maintaining the upper body stability of users with SCI.

Rowing speed and ergometer setup had a significant influence on foot force production in both able-bodied rowers and the rower with SCI. The able-bodied participants generated twice as much foot reaction force when rowing at a fast speed (40 SPM), a large knee RoM (90° or 120°), and in a forward seat position, compared to other conditions. Higher rowing speed causes greater acceleration, and a larger knee RoM allows the lower limb joints to generate more moment and power, both of which contributed to higher force at the foot. Larger knee RoM or faster speed, however, did not yield higher foot force or knee moment in the rower with SCI. This highlights the inherent difference in the behavior of electrically stimulated muscles and voluntarily contracted muscles. The FES stimulator delivers a fixed amount of energy to the muscle, which is either used to generate motion or transmitted through the leg until it reaches the foot stretcher, reflected as the foot reaction force. In other words, the foot reaction force could be compromised if a larger range of motion is to be completed. This does not apply to able-bodied rowers, who can adjust muscle recruitment depending on the task. To meet the more energy-demanding rowing conditions (i.e., rowing with a large knee RoM or a faster speed), able-bodied participants could recruit additional motor units within the given muscles or engage more muscles, which contributed to larger foot reaction forces. This also explains why the rower with SCI was not able to complete conditions that required fast speeds or a large knee RoM. Knowing the potential trade-off between producing force and producing motion in FES rowing, a clinical application to enhance force production and joint loading could be to statically load the lower limb using the FES on the adapted ergometer. Specifically, clinicians can fix the user’s feet on the foot stretcher, specify a fixed seat position and knee angle using the front and back stoppers, and isometrically contract the knee extensor and flexor using the FES. This configuration is similar to FES-induced standing but in a seated position, which allows for a more stable posture [34].

The rower with SCI generated the largest force and knee moment using the self-selected style, with the force magnitude (0.39 BW) approaching the upper limit of previously reported foot reaction forces in rowers with SCI (0.15–0.46 BW) [12,14,15]. This appeared to result from a combination of the rower setup, good hand–leg coordination, and an experienced and strong participant. The individual in the current study had rowed for six years, which made him more experienced than those included in previous studies. He also began rowing very shortly after experiencing a spinal cord injury and had no obvious leg muscle atrophy compared to many individuals with chronic SCI.

A common goal of FES rowing for individuals with SCI is to prevent bone loss by loading the lower extremities. FES during active-resisted standing generated 1.50 BW knee contact force in individuals with SCI, which significantly attenuated bone loss at the tibia and femur [34]. The individual with SCI in the present study received 1.21 to 2.23 BW compressive tibiofemoral force when rowing in various forms, suggesting that ergometer setup and speed are crucial factors in determining whether the exercise will be beneficial to the bone. Furthermore, the large variance in reported knee joint loading (1.25–4.6 BW) in previous FES rowing studies could be partially attributed to the difference in rowing forms and setups between participants [13,15]. The results show that the external loading (foot reaction force) was much smaller than the internal knee joint loading, suggesting that muscle contraction was the primary contributor to tibiofemoral force in FES rowing. Seat position would affect the muscle working range, and our data suggest that the effects of seat position on foot reaction force were different between able-bodied rowers and the rower with SCI: the former generated higher force at a forward seat position and the latter at a more rear position. This indicates that the optimum joint working range may exist differently for electrically stimulated muscles and voluntarily contracted muscles. Therefore, applying FES at specific knee ranges of motion could optimize muscle force generation and consequently maximize knee loading. The findings bring up an issue that warrants researchers to further investigate it with more rowers with SCI.

One limitation of the current study is that only the right foot reaction force was measured. We argue that rowing is symmetrical, especially when both legs are constrained by the knee stabilizer. A previous study that measured bilateral foot reaction forces in FES rowing reported a 7% difference between the left and right foot reaction force. Here, the kinematic data show nearly identical joint angles for the left and right legs. Another limitation is that we only tested on one participant with SCI, which limited the generalizability of the results. It is worth noting that SCI characteristics, such as injury level, severity, and time since injury, may affect each individual’s response to FES rowing, and further studies should include a heterogeneous cohort and take into account these factors. Although this participant does not represent all individuals with SCI, the data are important to inform future investigations on FES rowing biomechanics. In particular, this case study highlights that there may be a trade-off between producing force and producing motion in FES rowing due to the inability to recruit additional motor units and muscles in SCI users. In our musculoskeletal model of the SCI participant, we did not use subject-specific anthropometry or muscle properties due to the lack of full-body dual-energy X-ray absorptiometry (DXA) and dynamic ultrasound imaging data on this SCI participant. We did not consider any possibility of muscle spasm or abnormal firing patterns, which could affect knee loading. Thus far, there are no standard ways to measure muscle spasms, nor is there a muscle model that takes this factor into account. Future studies could validate muscle activation with EMG measurements. Our validation compared the muscle activation timing predicted by the musculoskeletal model to when the stimulator was pressed by the user, and it showed good agreement, which gives us confidence in the model predictions (Figure S2).

## 5. Conclusions

The design of the adapted ergometer, which constrained trunk and hip motion, limited the amount of force that could be produced during rowing. Changing ergometer setup affected force production for both able-bodied people and the individual with SCI, but the setup that yielded the highest foot reaction forces and knee moments was different for able-bodied rowers versus the rower with SCI. Able-bodied rowers adopted a fast rowing speed, forward seat position, and large knee RoM. In contrast, the rower with SCI had a rear seat position and smaller knee RoM. Our findings support future research with a larger cohort of heterogeneous SCI individuals and suggest rowing setups and forms to be considered during FES rowing at home or in clinical settings.

## Figures and Tables

**Table 1:** Target and achieved knee angle, range of motion (RoM), and rowing speed for each condition for the participant with spinal cord injury, and the normalized peak resultant foot reaction force, peak knee extension moment, and peak compressive tibiofemoral force for each condition. The shaded area indicates conditions in which the participant was unable to complete the rowing cycle. SPM: strokes per minute.

Target Knee Angle, RoM, and Speed (SPM)	Achieved Knee Angle, RoM, and Speed (SPM)	Peak Foot Reaction Force (BW)	Peak Knee Moment (Nm/kg)	Peak Tibiofemoral Force (BW)
self-selected	114–176°, 62°, 53	0.39	0.49	1.83
45–115°, 70°, 40	65–138°, 73°, 42	0.26	0.40	1.96
45–135°, 90°, 40	117–175°, 58°, 52			
45–115°, 70°, 35	63–130°, 67°, 31	0.28	0.41	1.21
45–135°, 90°, 35	71–179°, 108°, 29	0.27	0.36	1.97
45–165°, 120°, 35	146–175°, 29°, 27			
70–140°, 70°, 35	92–174°, 82°, 36	0.29	0.36	2.23
mean		0.3	0.4	1.81
maximum		0.39	0.49	2.23
minimum		0.26	0.36	1.21

**Table 2:** Mean (standard deviation) normalized peak foot reaction force and knee extension moment at three rowing speeds under two knee ranges of motion (minimum to maximum knee angle). BW: body weight, SPM: strokes per minute, RoM: range of motion, * indicates significant difference between 25 and 35 SPM, Ϯ indicates significant difference between 35 and 40 SPM, and § indicates significant difference between 25 and 40 SPM. *, Ϯ, §: *p* < 0.05.

Knee RoM	Speed (SPM)	Peak Foot Reaction Force (BW)	Peak Knee Moment (Nm/kg)
RoM 70° (45–115°)	25 SPM	0.26 (0.06) *	0.29 (0.14) *
	35 SPM	0.36 (0.06) ^Ϯ^	0.54 (0.24)
	40 SPM	0.42 (0.08) ^§^	0.63 (0.32) ^§^
RoM 90° (45–135°)	25 SPM	0.29 (0.07) *	0.34 (0.15) *
	35 SPM	0.39 (0.07) ^Ϯ^	0.63 (0.26) ^Ϯ^
	40 SPM	0.45 (0.08) ^§^	0.71 (0.31) ^§^

**Table 3:** Mean (standard deviation) normalized peak foot reaction force and knee extension moment when rowing under three knee ranges of motion (RoM) at two speeds. BW: body weight, SPM: strokes per minute, RoM: range of motion; * indicates significant difference between RoM 70° and RoM 90°, and § indicates significant difference between RoM 70° and RoM 120°. *, §: *p* < 0.05.

Speed (SPM)	Knee RoM	Peak Foot Reaction Force (BW)	Peak Knee Moment (Nm/kg)
25 SPM	RoM 70°	0.26 (0.06) *	0.29 (0.14) *
	RoM 90°	0.29 (0.07)	0.34 (0.15)
	RoM 120°	0.30 (0.07) ^§^	0.39 (0.26) ^§^
35 SPM	RoM 70°	0.36 (0.06) *	0.54 (0.24) *
	RoM 90°	0.39 (0.07)	0.63 (0.26)
	RoM 120°	0.41 (0.08) ^§^	0.61 (0.27) ^§^

**Table 4:** Mean (standard deviation) normalized peak foot reaction force and knee extension moment when rowing with three seat positions at speeds of 25 and 35 strokes per minute (SPM). Forward, middle, and rear seat positions indicate knee ranges of motion (RoM) of 45–115°, 70–140°, and 95–165°, respectively. BW: body weight, * indicates significant difference between forward and middle positions, Ϯ indicates significant difference between middle and rear positions, and § indicates significant difference between forward and rear positions. *, Ϯ, §: *p* < 0.05.

Speed (SPM)	Seat Position	Peak Foot Reaction Force (BW)	Peak Knee Moment (Nm/kg)
25 SPM	Forward	0.26 (0.06)	0.29 (0.14) *
	Middle	0.24 (0.07)	0.19 (0.14)
	Rear	0.22 (0.07) ^§^	0.14 (0.12) ^§^
35 SPM	Forward	0.36 (0.06)	0.54 (0.24) *
	Middle	0.33 (0.08)	0.34 (0.19) ^Ϯ^
	Rear	0.29 (0.07) ^§^	0.22 (0.14) ^§^

## Data Availability

The original data presented in the study are openly available in FigShare at https://figshare.com/articles/dataset/dx_doi_org_10_6084_m9_figshare_27969279/27969279 (accessed on 1 December 2024).

## Supplementary Materials

The following supporting information can be downloaded at: https://www.mdpi.com/article/10.3390/bioengineering12010075/s1, Figure S1: Anteroposterior (AP) and vertical pelvis force during self-selected rowing condition of the participant with spinal cord injury. Forces were calculated using (1) dynamic analysis considering measured external forces and (2) residual reduction algorithm (RRA) in OPENSIM. The two methods reached reasonable agreement; Figure S2: Synchronized data showing the time difference between hand switch signal and estimated muscle force from OPENSIM. The timing of muscle activation predicted by static optimization in OPENSIM matches the timing of switch pressing and releasing by the participant. On average, the quadriceps muscle was activated ~150 ms after the participant pressed the switch, and the hamstrings was activated ~50 ms before the participant completely released the switch. Table S1: Ergometer setup and rowing speed for each condition and mean (standard deviation) normalized peak hand pulling force and rear impact force of each condition. SPM: strokes per minute. BW: body weight.
