# Supplementary material for: Effect of Adapted Ergometer Setup and Rowing Speed on Lower Extremity Loading in People with and Without Spinal Cord Injury
Source: Bioengineering (Basel). 2025 Jan 15;12(1):75. doi: 10.3390/bioengineering12010075 (PMC11761565; doi:10.3390/bioengineering12010075)
Supplement: Supplementary file 1 [file bioengineering-12-00075-s001.zip › bioengineering-3382247-supplementary.pdf]

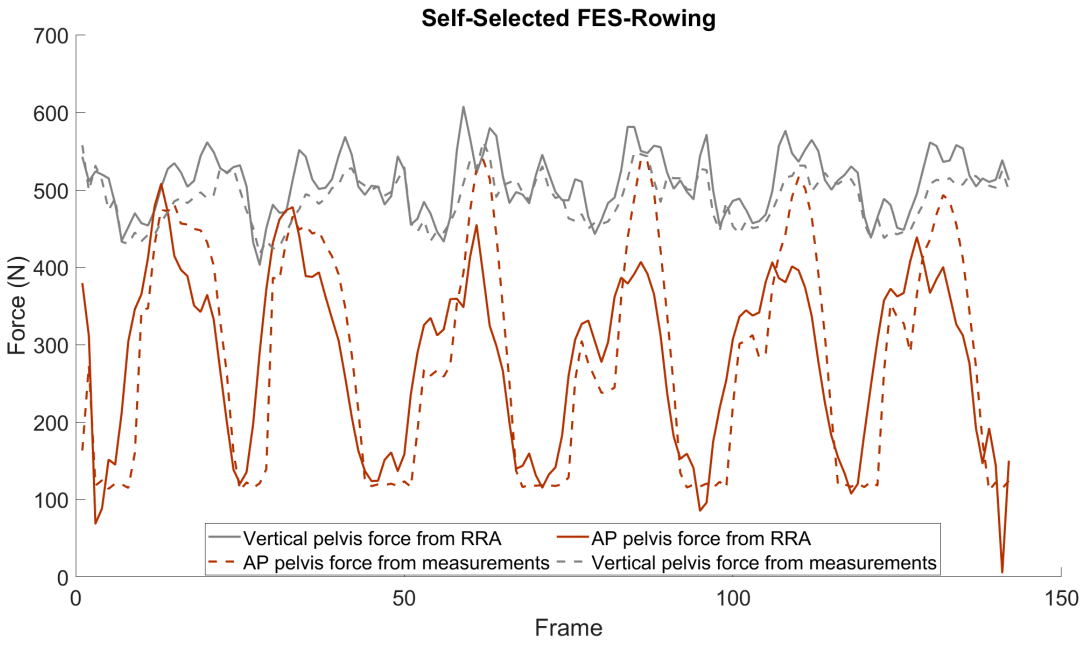

Figure S1: Anteroposterior (AP) and vertical pelvis force during self-selected rowing condition of the participant with spinal cord injury. Forces were calculated using 1) dynamic analysis considering measured external forces and 2) residual reduction algorithm (RRA) in OPENSIM. The two methods reached reasonable agreement.

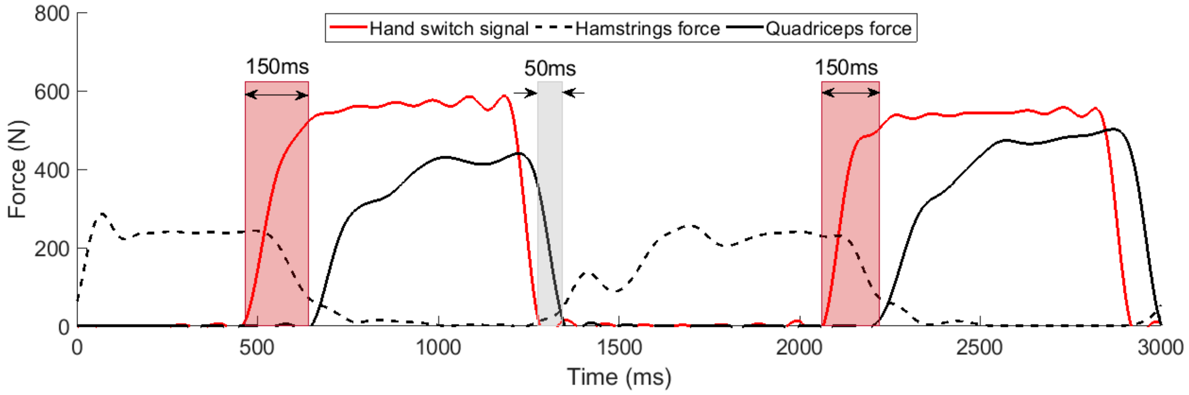

Figure S2: Synchronized data showing the time difference between hand switch signal and estimated muscle force from OPENSIM. The timing of muscle activation predicted by static optimization in OPENSIM matches the timing of switch pressing and releasing by the participant. On average, the quadriceps muscle was activated ~150 ms after the participant pressed the switch, and the hamstrings was activated ~50 ms before the participant completely released the switch.

Table S1. Ergometer setup and rowing speed for each condition, and mean (standard deviation) normalized peak hand pulling force and rear impact force of each condition. SPM: strokes per minute. BW: body weight.

**Condition**	**Knee Range of Motion**	**Speed (SPM)**	**Seat Position**	**Hand Pulling Force (BW)**	**Rear Impact Force (BW)**
1	45°-115°	40	Forward	0.47 (0.18)	0.43 (0.20)
2	45°-135°	40	Forward	0.51 (0.18)	0.36 (0.18)
3	45°-115°	25	Forward	0.28 (0.16)	0.27 (0.16)
4	45°-115°	35	Forward	0.40 (0.15)	0.35 (0.18)
5	45°-135°	25	Forward	0.31 (0.15)	0.24 (0.13)
6	45°-135°	35	Forward	0.44 (0.16)	0.31 (0.13)
7	45°-165°	25	Forward	0.33 (0.14)	0.17 (0.10)
8	45°-165°	35	Forward	0.48 (0.20)	0.19 (0.10)
9	70°-140°	25	Middle	0.25 (0.14)	0.21 (0.13)
10	70°-140°	35	Middle	0.35 (0.13)	0.26 (0.15)
11	95°-165°	25	Rear	0.23 (0.14)	0.17 (0.10)
12	95°-165°	35	Rear	0.32 (0.13)	0.18 (0.11)
